# Investigating the Anti-Inflammatory, Analgesic, and Chondroprotective Effects of *Gynostemma pentaphyllum* (Thunb.) Makino in Osteoarthritis: An In Vitro and In Vivo Study

**DOI:** 10.3390/ijms25179594

**Published:** 2024-09-04

**Authors:** Hee-Geun Jo, Chae Yun Baek, Yeseul Hwang, Eunhye Baek, Chanyoon Park, Ho Sueb Song, Donghun Lee

**Affiliations:** 1 Department of Herbal Pharmacology, College of Korean Medicine, Gachon University, 1342 Seongnamdae-ro, Sujeong-gu, Seongnam-si 13120, Republic of Korea; jho3366@hanmail.net (H.-G.J.); cyning20@gachon.ac.kr (C.Y.B.);; 2 Naturalis Inc., 6 Daewangpangyo-ro, Bundang-gu, Seongnam-si 13549, Republic of Korea; 3 RexSoft Inc., 1 Gwanak-ro, Gwanak-gu, Seoul 08826, Republic of Korea; 4 Department of Acupuncture & Moxibustion Medicine, College of Korean Medicine, Gachon University, 1342 Seongnamdae-ro, Sujeong-gu, Seongnam-si 13120, Republic of Korea

**Keywords:** *Gynostemma pentaphyllum* (Thunb.) Makino, osteoarthritis, anti-inflammatory, chondroprotective, analgesic, herbal medicine

## Abstract

Osteoarthritis (OA) is an age-related disease characterized by inflammation, pain, articular cartilage damage, synovitis, and irreversible disability. *Gynostemma pentaphyllum* (Thunb.) Makino (GP), a herbal medicine traditionally used in East Asia for its anti-inflammatory properties, was investigated for its potential to modulate OA pathology and symptoms. This study evaluated GP’s efficacy in inhibiting pain, functional decline, and cartilage destruction in monosodium iodoacetate-induced OA and acetic acid-induced writhing models. Additionally, the effects of GP on OA-related inflammatory targets were assessed via mRNA and protein expression in rat knee cartilage and lipopolysaccharide-induced RAW 264.7 cells. The GP group demonstrated significant pain relief, functional improvement, and cartilage protection. Notably, GP inhibited key inflammatory mediators, including interleukin (IL)-1β, IL-6, matrix metalloproteinases (MMP)-3 and MMP-13, cyclooxygenase-2, and prostaglandin E receptor 2, surpassing the effects of active controls. These findings suggest that GP is a promising candidate for disease-modifying OA drugs and warrants further comprehensive studies.

## 1. Introduction

Osteoarthritis (OA) is a prevalent age-related disease characterized by chronic pain, articular cartilage damage, synovitis, and irreversible disability [1]. Over the past 30 years, the healthcare burden of OA has increased due to global aging, making it a major public health issue [2,3]. Countries with higher sociodemographic indices have a disproportionately high burden of OA, exacerbating health issues [4]. Despite this urgency, the response to OA is hindered by the limited understanding of its pathology. Historically, OA has been attributed to degenerative changes caused by cartilage wear and tear. However, emerging research indicates that low-intensity systemic and local musculoskeletal inflammation significantly contributes to the pathogenesis of OA [5,6]. Age-related cellular senescence and the secretion of senescence-associated secretory phenotypic (SASP) factors, including inflammatory cytokines, chemokines, and enzymes, are directly involved [7,8,9,10]. Pro-inflammatory factors such as interleukin (IL)-1β, IL-6, and tumor necrosis factor (TNF)-α induce chondrocytes and synovial fibroblasts to produce chondrolytic enzymes like matrix metalloproteinases (MMPs) and additional pro-inflammatory factors, exacerbating OA [6,11]. Therefore, developing therapeutic agents that inhibit the chronic inflammation underlying OA to reverse pain, dysfunction, and disease progression is urgently needed.

Given the complexity of OA pathology, which involves numerous factors, no cure for OA is currently available [12,13]. Various viscosity supplements have been developed to prevent progressive cartilage destruction, but their efficacy and safety remain highly controversial [14]. Therefore, the clinical management of OA focuses on symptomatic relief, primarily through non-drug approaches such as appropriate exercise, weight loss, and lifestyle changes, as recommended by most clinical guidelines [15]. Nonsteroidal anti-inflammatory drugs (NSAIDs) and glucocorticoids are widely used to manage pain in OA. However, NSAIDs are associated with adverse events including gastrointestinal, cardiovascular, and renal issues, raising concerns about their long-term safety and efficacy [16,17,18]. Glucocorticoids, which are commonly used, have not shown any notable advantages over physical therapy in well-designed clinical trials and can cause severe complications such as steroid-induced osteonecrosis [19,20]. Consequently, the development of novel OA therapeutics that can overcome the safety and efficacy limitations of the current treatments and delay disease progression is critical. Despite this urgency, none of the drugs classified as disease-modifying OA drugs (DMOADs) has demonstrated efficacy in large-scale clinical trials or has secured regulatory approval [21,22]. Therefore, ongoing studies need to explore new drug candidates and optimize the therapeutic mechanisms for OA.

In light of the above context, the search for natural product-based disease-modifying osteoarthritis drugs (DMOADs) has intensified, focusing on their ability to modulate multiple inflammation-related pathways through unique multicomponent and multitargeted actions [23,24]. Recently, studies have explored the potential effects of East Asian herbal medicine (EAHM) on various inflammatory musculoskeletal diseases, including OA, rheumatoid arthritis, and psoriatic arthritis [25]. EAHM encompasses medicinal herbs listed in the pharmacopoeias of Korea, China, and Japan and is a valuable resource for discovering DMOAD candidates because of its widespread use by hundreds of millions of people [26,27,28,29,30,31,32]. Among the numerous EAHMs, *Gynostemma pentaphyllum* (Thunb.) Makino (GP) is a promising herb with broad-spectrum pharmacological activities. These include potent anti-inflammatory, antioxidant, anticancer, hepatoprotective, hypoglycemic, antidementia, and anti-Parkinsonian benefits, lipid modulation, and inhibition of ischemic injury [33]. It influences key signaling pathways such as nuclear factor-κB (NF-κB), mitogen-activated protein kinase (MAPK), phosphoinositide 3-kinase (PI3K)/Akt, transducer and activator of transcription 1 (STAT1), and peroxisome proliferator-activated receptor gamma (PPAR-γ), among others. These pathways suggest that GP is a promising candidate [34]. However, its anti-OA activity has been minimally studied, leaving uncertainty as to whether GP merits further investigation as a DMOAD candidate.

Therefore, we conducted this screening study to evaluate whether GP is a potential candidate as a DMOAD. We focused on its ability to simultaneously modulate OA-associated pain, functional loss, cartilage destruction, and overall inflammatory pathology. In this study, we observed multi-targeted inhibition of inflammation-related factors, such as cytokines, in an in vitro model. Additionally, in an in vivo model, we assessed whether GP could inhibit pain, disability, and cartilage destruction in patients with OA.

## 2. Results

### 2.1. HPLC Analysis

**Figure 1:**
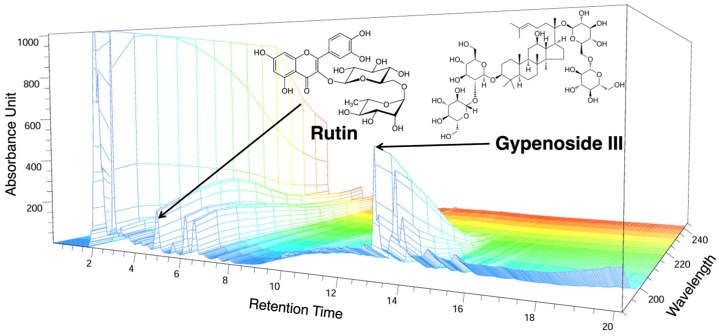
HPLC chromatogram of the GP extract at 203 nm: rutin and gypenoside III retention time = 5.00 min and 13.235 min. The *x*-axis shown the retention time; the *y*-axis shown the absorbance unit; the *z*-axis indicates the absorbance unit. HPLC: high-performance liquid chromatography, GP: *Gynostemma pentaphyllum* (Thunb.).

In the present study, we identified rutin and ginsenoside III in GP using HPLC-UV spectroscopy. The extract contained 23.1093 mg/g rutin and 52.9241 mg/g gypenoside III. Figure 1 shows the HPLC chromatograms and the chemical structures of the compounds.

### 2.2. Effect on AAW Reponses

**Figure 2:**
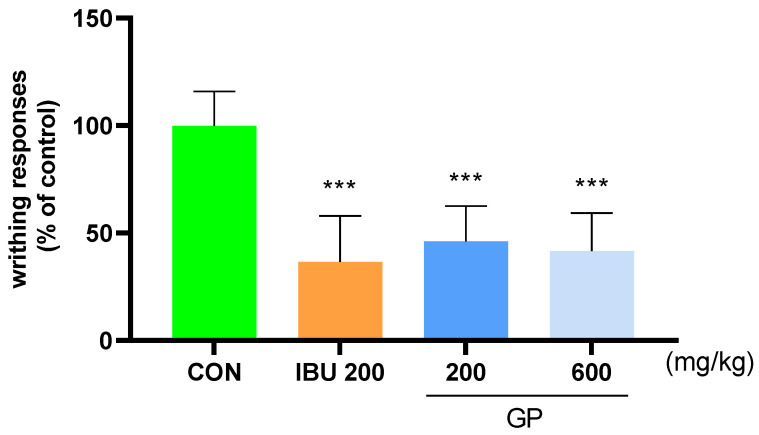
Number of writhing responses in AAW ICR mice. After 30 min of sample treatment, groups were treated with 0.7% acetic acid (i.p.) before 10 min measuring. *** *p* < 0.001 vs. CON using one-way ANOVA, Dunnett’s test. AAW: acetic acid-induced writhing, CON: control, GP: *Gynostemma pentaphyllum*, IBU 200: ibuprofen 200 mg/kg.

To determine the degree of pain improvement, the analgesic effect of GP was investigated based on the writhing response of the mice injected with acetic acid. The results of the writhing response of the mice injected with acetic acid showed that after 10 min, the average writhing in the CON group was 100%. The IBU 200 group had an average of 36.57% and the GP 600 group had an average of 41.75%, indicating that GP 600 was more effective than IBU 200 (Figure 2).

### 2.3. Analgesic Effects on MIA-Induced OA Model

**Figure 3:**
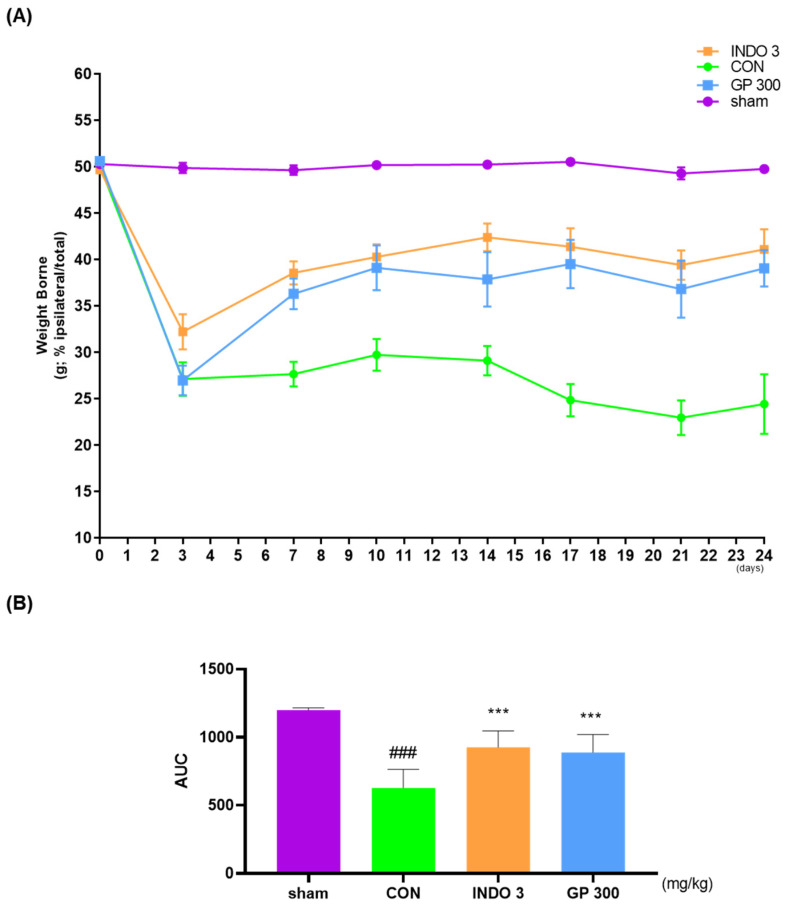
The effects of GP on weight-bearing of hind limb in MIA-induced OA model. (**A**) Weight-bearing distribution of MIA rats on 0–24 days with GP 300 or INDO 3 treatment and (**B**) AUC were analyzed by incapacitance meter tester. ^###^ *p* < 0.001 vs. sham, *** *p* < 0.001 vs. CON. AUC: area under the curve, GP: *Gynostemma pentaphyllum*, INDO 3: indomethacin 3 mg/kg, MIA: monosodium iodoacetate.

Hind limb weight-bearing is a marker of joint pain, and discomfort is commonly utilized in animal models to evaluate the analgesic effects of OA samples. The weight-bearing ratio was measured for 24 d from day 0. As illustrated in Figure 3A, the weight-bearing ratio in the CON group significantly decreased on day 3 and was lower than that in the sham group. In contrast, the GP 300 group showed a significant improvement in weight bearing compared to the CON group. In particular, the improvement in weight-bearing by the GP 300 group was comparable to that in the INDO 3 group (Figure 3B).

### 2.4. Improving Effects of GP on Joint Cartilage Damage in OA Rat Model

**Figure 4:**
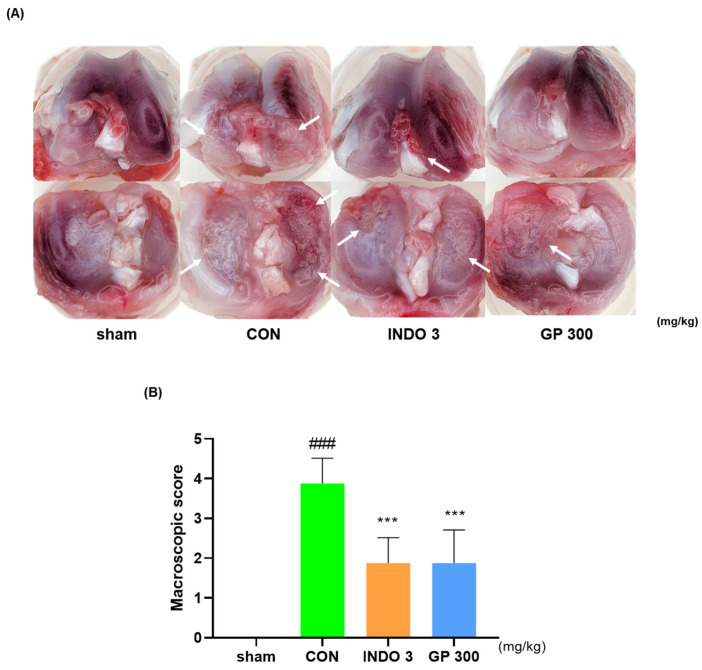
Photographs of the knee joint cartilages of OA rats. OA rats were administrated INDO 3 and GP 300. (**A**) Representative photo in knee joint cartilage of OA rat. The arrows signed the cartilage erosion point. (**B**) The scoring of macroscopic. ^###^ *p* < 0.001 vs. sham, *** *p* < 0.001 vs. CON by a one-way analysis, Dunnett’s test. GP: *Gynostemma pentaphyllum*, INDO 3: indomethacin 3 mg/kg, MIA: monosodium iodoacetate.

A representative image of the right knee joints in each group verified that GP 300 prohibited knee joint cartilage damage-induced OA. As shown in Figure 4A, the joint cartilage of the sham group was lustrous and smooth. In contrast, the cartilage in the CON group was less shiny and rougher, with evidence of damage in some areas. The MIA-induced cartilage erosion was significantly reduced in the rats administered GP 300 and INDO 3. According to the macroscopic score, the degree of cartilage erosion in the experimental groups treated with AG and INDO 3 was significantly reduced (Figure 4B). Specifically, the effectiveness of GP 300 in preventing cartilage erosion was compared to that of INDO 3.

### 2.5. Effects of GP on Inflammatory Cytokines in OA Rat Model

**Figure 5:**
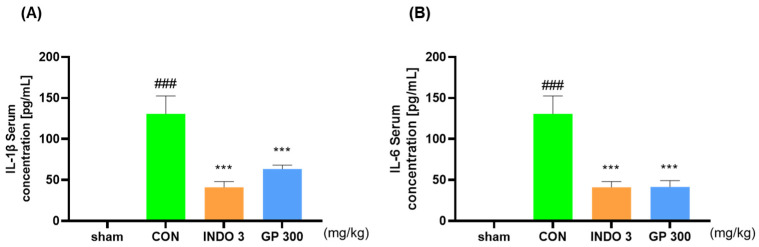
Expression levels of (**A**) IL-1β and (**B**) IL-6 levels in serum from OA rats. Rats were treated with INDO 3 and GP 300 for 24 d. ^###^ *p* < 0.001 vs. sham, *** *p* < 0.001 vs. CON by one-way ANOVA, Dunnett’s test. GP: *Gynostemma pentaphyllum*, INDO 3: indomethacin 3 mg/kg, MIA: monosodium iodoacetate.

The expression levels of IL-1β and IL-6 were evaluated after separating serum in experimental group. The GP 300 group showed significantly reduced levels of IL-1β and IL-6 in the serum compared with the CON group. In particular, the results for the GP 300 group indicated a reduction in cytokine levels comparable to those observed in the INDO 3 group (Figure 5A,B).

### 2.6. Effects of GP on Cytokine Responses in Cartilage

**Figure 6:**
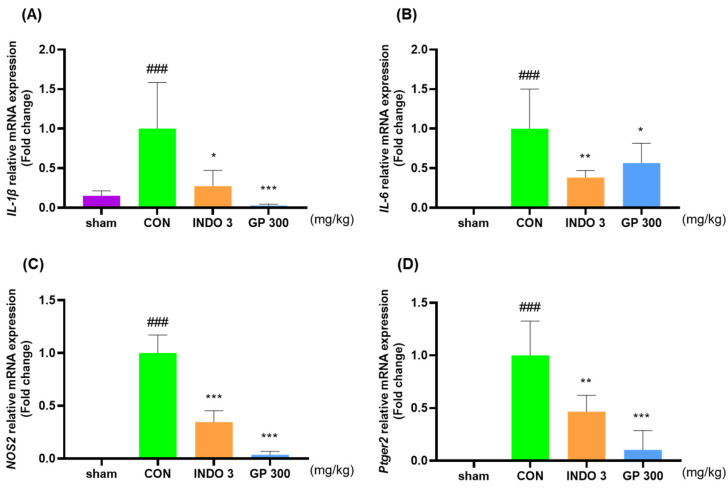

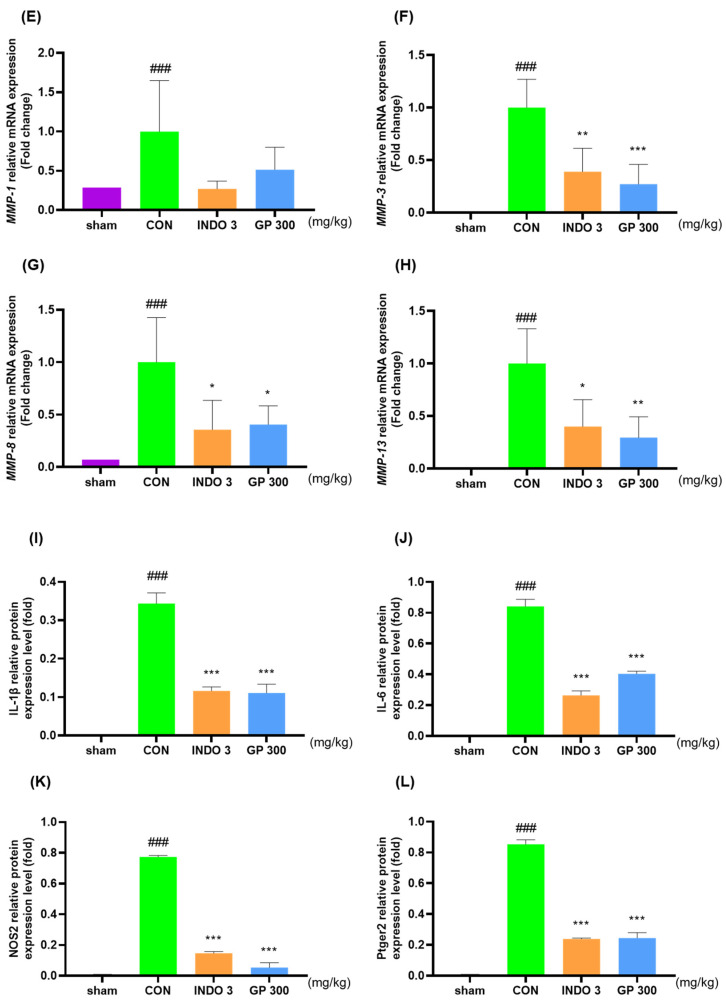

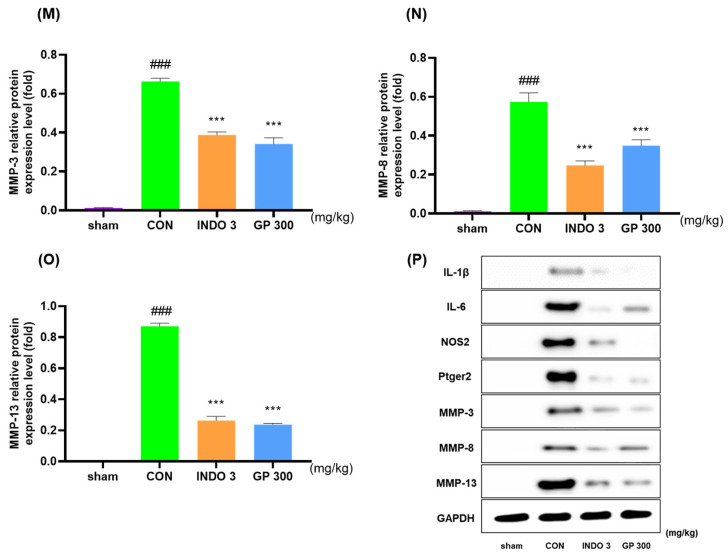
Evaluation of cytokine level in cartilage tissue in experimental groups. (**A**–**H**) mRNA expression of *IL-1β*, *IL-6*, *NOS2*, *Ptger2*, *MMP-1*, *MMP-3*, *MMP-8*, and *MMP-13* measured using qRT-PCR. (**I**–**P**) Protein expression of IL-1β, IL-6, NOS2, Ptger2, MMP-3, MMP-8, and MMP-13 measured with western blot analysis. * *p* < 0.05 vs. CON, ** *p* < 0.01 vs. CON, *** *p* < 0.001 vs. CON, ^###^ *p* < 0.001 vs. sham by one-way ANOVA, Dunnett’s test. GP: *Gynostemma pentaphyllum*, INDO 3: indomethacin 3 mg/kg, MIA: monosodium iodoacetate.

Measurement of the mRNA expression levels of *IL-1β*, *IL-6*, *NOS2*, *Ptger2*, *MMP-1*, *MMP-3*, *MMP-8*, and *MMP-13* in the OA rats showed that the GP 300 group showed significantly decreased levels of *IL-1β*, *IL-6*, *NOS2*, *Ptger2*, *MMP-1*, *MMP-3*, *MMP-8*, and *MMP-13* in the cartilage tissue (Figure 6A–H) compared with the CON group. In particularly, the GP 300 group had lower levels of *IL-1β*, *IL-6*, *NOS2*, *Ptger2*, *MMP-1*, *MMP-3*, *MMP-8,* and *MMP-13* than the INDO 3 group. Western blotting demonstrated the downregulating effects of GP on IL-1β, IL-6, NOS2, Ptger2, MMP-3, MMP-8, and MMP-13 in OA rats (Figure 6I–P).

### 2.7. Effects of GP on Cytokine Responses in LPS-Treated RAW264.7 Cells

**Figure 7:**
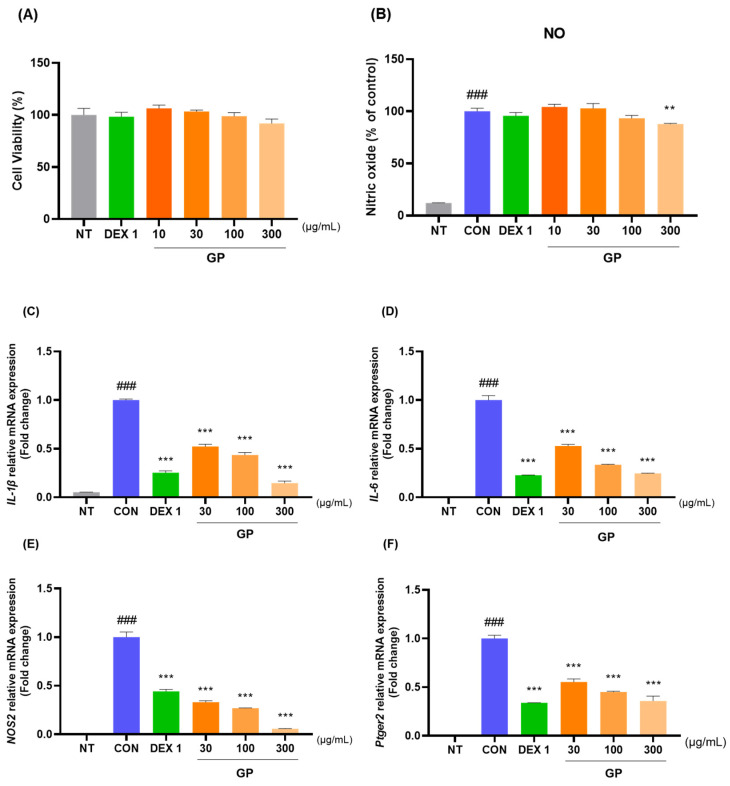

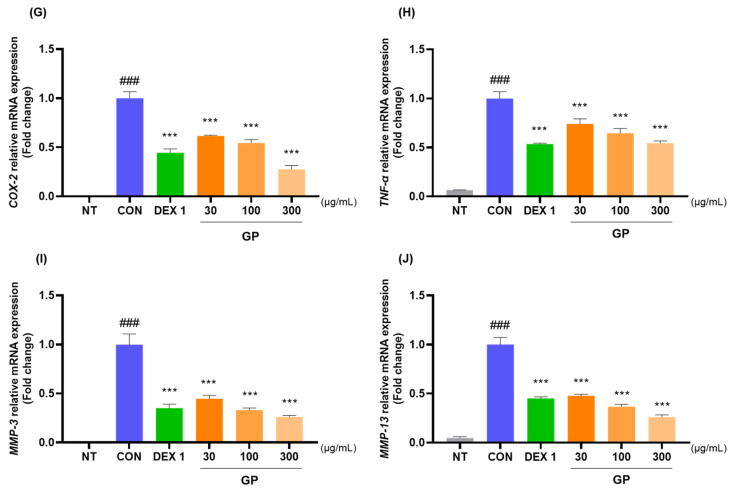

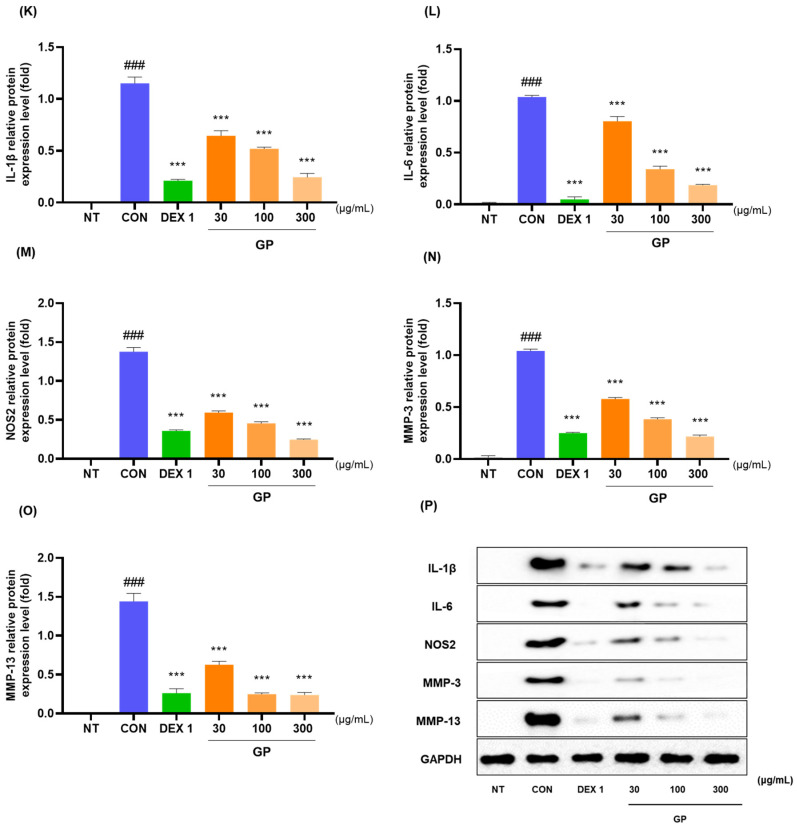
Effects of GP on (**A**) cell viability and (**B**) NO generation in LPS-treated RAW264.7 cells, the mRNA expression level of (**C**–**J**) *IL-1β*, *IL-6*, *NOS2*, *Ptger2*, *COX-2*, *TNF-α*, *MMP-3*, and *MMP-13* and the protein expression level of (**K**–**P**) IL-1β, IL-6, NOS2, MMP-3, and MMP-13 in RAW264.7 cells. Cell were treated with GP (30, 100, and 300 µg/mL) and 500 ng/mL LPS during 24 h. ^###^ *p* < 0.001 vs. sham, ** *p* < 0.01 vs. CON, *** *p* < 0.001 vs. CON by one-way ANOVA, Dunnett’s test. CON: control, DEX 1: dexamethasone 1 µg/mL, GP: *Gynostemma pentaphyllum*, LPS: lipopolysaccharide, NT: non-treated.

The anti-inflammatory effects of GP were evaluated in the LPS-treated RAW264.7 cells. The GP indicated anti-inflammatory effects by decreasing NO levels, mRNA expression of IL-1β, IL-6, NOS2, Ptger2, COX-2, TNF-α, MMP-3, and MMP-13, and protein expression of IL-1β, IL-6, NOS2, MMP-3, and MMP-13. No latent cytotoxicity of the GP up to 300 µg/mL was detected in the RAW264.7 cells (Figure 7A). NO production was dose-dependently reduced in the LPS-treated RAW264.7 cells by the GP. In particular, approximately 20% NO reduction was observed with GP 300 compared to CON (Figure 7B). The anti-inflammatory effects of the GP on the LPS-treated RAW264.7 cells were evaluated by qRT-PCR and western blotting. As illustrated in Figure 7C–P, the mRNA and protein expression level of pro-inflammatory cytokines such as *IL-1β*, *IL-6*, *NOS2*, *Ptger2*, *COX-2*, *TNF-α*, *MMP-3*, and *MMP-13* in mRNA (Figure 7C–J) and IL-1β, IL-6, NOS2, MMP-3, and MMP-13 in protein (Figure 7K–P) were suppressed by the GP treatment in the LPS-stimulated RAW264.7 cells. Based on the analysis of mRNA expression levels and Western blot images, GP was found to dose-dependently decrease the expression of IL-1β, IL-6, NOS2, MMP-3, and MMP-13. Notably, the GP 300 group exhibited anti-inflammatory effects on all these cytokines compared to DEX 1.

## 3. Discussion

As demonstrated in the results, GP exhibited consistent anti-OA effects in an in vivo model. Additionally, it significantly inhibited inflammation-related markers such as IL-1β, IL-6, NOS2, MMP-3, and MMP-13 in the cartilage of OA-induced animal models and LPS-stimulated RAW 264.7 cells. These effects were statistically superior to those of the active control, indomethacin, and dexamethasone and were observed in a dose-dependent manner. To the best of our knowledge, this is the first study to evaluate the potential of GP as a DMOAD candidate based on its ability to inhibit various OA symptoms and its effects on a broad spectrum of inflammatory targets associated with OA. The implications of these results in light of previous studies are significant and require further investigation.

In this study, we identified gypenoside III (PubChem CID: 9898279) and rutin (PubChem CID: 5280805) as the main active components of GP, as confirmed by HPLC-UV analysis. Gypenoside III is the most prevalent compound among the ginsenoside and has been extensively studied for its pharmacokinetic properties, particularly in central nervous system diseases [35]. It exhibits pronounced anti-inflammatory activity by downregulating IL-1β and TNF-α and preventing the production of pro-inflammatory factors through COX-2 and NF-κB control mechanisms [36]. Additionally, gypenoside III has antioxidant effects, mitigating oxidative stress in mitochondria via a reactive oxygen species regulatory mechanism [37]. These bioactivities are significant because the mitochondria are a key target of this compound, and several studies have demonstrated the modulatory effects of gypenoside III on mitochondria-related pathologies. Anti-inflammatory and antioxidant activities are critical for the development of inhibitors of cartilage senescence and apoptosis in OA. Recent studies have explored candidate drugs targeting these pathways, recognizing that homeostatic management of mitochondria may contribute to the pathogenesis of OA and inhibit cartilage damage [38,39]. Based on these findings, the broad anti-inflammatory activity and symptomatic suppression of OA exhibited by GP may be attributed to its mechanism of action. Rutin has also demonstrated inhibitory properties against various chronic inflammatory pathologies by reducing levels of pro-inflammatory markers such as IL-1β, IL-6, COX2, and TNF-α while exhibiting potent antioxidant activity [40]. Interestingly, unlike gypenoside III, rutin significantly improves inflammatory cell infiltration, cartilage and bone erosion, and synovial hyperplasia in rheumatoid arthritis models by lowering IL-1β and TNF-α and inhibiting the NF-κB pathway [41]. These findings are consistent with the observations of the present study. However, it is important to note that this study aimed to explore the potential of GP as a whole extract for the treatment of OA by leveraging its multicomponent and multitarget modulation capabilities. Therefore, the major compounds identified by HPLC alone cannot fully explain the results of this study. GP contains hundreds of saponins such as gypenosides, and research in this area is ongoing [42,43]. Identifying the specific components underlying the anti-OA effects observed in this study will be crucial for future research.

In the in vivo study, we used the monosodium iodoacetate (MIA) injection model to assess the anti-OA efficacy of GP and the acetic acid-induced writhing model to explore its analgesic effects. The MIA model is well established for its validity and reliability in mimicking OA pathology by inducing chondrocyte death and inflammation, thus replicating advanced OA [44]. In our study, GP inhibited painful dysfunction and cartilage destruction in this model at levels comparable to those of active control NSAIDs. Additionally, the analgesic effects were observed in a dose-dependent manner in the writhing model, providing quantitative cross-validation of the results. GP significantly reduced the serum cytokine levels of IL-1β and IL-6 in these animals. Similar trends were observed in the cytokine response of knee joint cartilage in the MIA animal model, where the inhibition of IL-1β, NOS2, and MMP-3 was significantly greater than that of indomethacin. The production of cytokines such as IL-1β, IL-6, and TNF-α is strongly associated with the NF-κB signaling pathway [45]. The activation intensity of this pathway in OA models correlates with disease worsening or improvement. Numerous anti-OA effects of natural products mediated by NF-κB pathway inhibition have been reported. Therefore, the results observed in the MIA model suggest that GP’s anti-OA effects are likely mediated by the strong anti-inflammatory activity associated with NF-κB pathway inhibition [31].

Consistent modulation of inflammatory targets was observed in the in vitro experiments in this study, supporting the results observed in the in vivo model. Notably, GP demonstrated greater potency than the active control, dexamethasone, in inhibiting NOS2 mRNA and relative protein expression of proinflammatory cytokines. A recent study reported that debris generated from cartilage breakdown triggers activation of Toll-like receptor 2 (TLR2), leading to the upregulation of NOS2 expression [46]. The TLR2-NOS axis impairs chondrocyte mitochondrial function and downregulates matrix protein expression, resulting in an inflammatory cartilage phenotype that promotes OA progression. This study also indicated that NOS2 inhibition could partially restore impaired mitochondrial function and adenosine triphosphate production. Given these findings, the NOS2 inhibitory activity of GP observed in this study likely contributes to the anti-OA effects observed in animal models. Additionally, GP’s inhibitory activity against Ptger2 was significant in both the animal knee joint cartilage and RAW264.7 cells. Previous studies have reported that stimulation of Ptger by prostaglandin E2 (PGE2) disrupts articular cartilage homeostasis and is directly related to OA pathophysiology, including elevated expression of pain-related molecules, such as IL-6 [47]. Subsequent research has shown that the selective inhibition of Ptger2 can delay abnormal subchondral bone formation and cartilage degeneration in OA [48]. Furthermore, GP demonstrated remarkable dose-dependent effects on MMP-3 and MMP-13, both of which are directly involved in the inhibition of progressive joint destruction in OA and are key targets for DMOAD development [49]. MMP-3 is significantly correlated with the severity of knee OA and MMP-13 drives cartilage degradation, making its inhibition an established anti-OA research focus [50,51]. Collectively, these findings suggest that the GP activity observed in vitro is not merely anti-inflammatory but supports a multitarget effect on the complex OA phenotype, including pain, functional impairment, and cartilage destruction. Therefore, the key targets of GP’s anti-OA activity and the active ingredients involved merit further in-depth studies. 

This study was conducted as an initial screening to determine whether GP is a viable candidate for the development of DMOADs. Further studies are required to reach definitive conclusions regarding the anti-OA activity of GP. First, the indications and activities of GP span a wide range and its active ingredients have not been fully characterized. In-depth characterization of the key components and targets is crucial for clarifying the value of GP, especially as a DMOAD candidate. We are preparing a follow-up study to expand these findings using network pharmacology techniques and advanced experimental methodologies. Second, although some of the effects of GP observed in this study were statistically significant, they did not surpass those of active control in all instances. This phenomenon is postulated to be dose-related. Given the dose-dependent nature of many targets, further studies are needed to determine the optimal dose for GP’s anti-OA activity. Moreover, GP is a relatively safe substance, with extensive pharmaceutical use and no reported toxicity issues [52]. However, studies on its pharmacokinetic properties, such as absorption, distribution, metabolism, and excretion, are necessary for its use in long-term OA treatment. In summary, although this study provided promising initial data, comprehensive future research is essential to fully establish GP’s potential as a DMOAD candidates.

## 4. Materials and Methods

All the experiments conducted in the present study were in accordance with the ARRIVE 2.0 guidelines [53]. Furthermore, the unprocessed western blot results for all the cell lines and additional assay markers can be found in Supplementary Materials.

### 4.1. Preparation of Gynostemma Pentaphyllum Extract (GP)

The leaves of *Gynostemma pentaphyllum* (Thunb.) Makino were purchased from Yaksudang Pharmaceutical, Ltd. (Seoul, Republic of Korea). These were identified by Professor Donghun Lee and a voucher specimen (D211117001) was deposited at the Department of Herbal Pharmacology, College of Korean Medicine, Gachon University. Extracts from the dried leaves were obtained using a reflux apparatus with 30% ethanol for 3 h at 85 °C, achieving an extract to herbal product ratio of 10:1. The extract was filtered a 90 mm filter paper (ADVANTEC Ltd., Tokyo, Japan), concentrated under reduced pressure using a rotary evaporator (Heidolph Instruments Inc., Schwabach, Germany), and freeze-dried (Sunileyela Ltd., Seongnam-si, Republic of Korea) to yield a 19.43% powder.

### 4.2. Analysis of GP Using High-Performance Liquid Chromatography (HPLC)

For the ingredient analysis of GP, chromatographic analysis was performed using an Agilent HPLC system (1100 series; Agilent, Santa Clara, CA, USA). The conditions for the HPLC analysis are listed in Table 1. A 10 mg sample of GP was dissolved in 1 mL of 50% methanol by sonication for 10 min.

### 4.3. Animals

Male Sprague (SD) rats (190–210 g) were used as the osteoarthritis (OA) induction model, and male ICR mice (30–40 g) were used as the writhing test model. The animals were supplied by DBL Inc. A total of 45 rats were used for the OA model and 40 mice were used for the acetic acid-induced writhing (AAW) model. The animals were acclimatized for at least 7 d in a regulated environment (temperature: 22 ± 2 °C, humidity: 55 ± 10%, 12 h light/dark cycle). Food and water were provided ad libitum. All the animal procedures were conducted in strict accordance with the Animal Care and Use Policy of Gachon University (GU1-2022-IA0071-01).

### 4.4. AAW Response

The ICR mice were randomly distributed into four groups and treated with control (CON), IBU 200 (Sigma, St. Louis, MO, USA), GP 200, or GP 600 (Table 2). Ibuprofen was used as the positive control. First, the mice were fed the samples and injected with 0.7% acetic acid 30 min after the sample treatment. Writhing responses were recorded after 10 min. The mice were allowed to show a writhing response for that period, and were measured for 10 min.

### 4.5. Design of OA Model and Sample Treatment

To establish the OA rat model, we designed an OA model for monosodium iodoacetate (MIA) induction. The groups were configured as sham, control (CON), indomethacin-treated (INDO 3; Sigma), and GP 300. The CON, INDO 3, and GP 300 groups had 50 μL of MIA solution (Sigma, St. Louis, MO, USA) injected into the right knee joint capacity to make the OA models (Table 3). All the groups had the samples administered orally daily for 24 d.

### 4.6. Weight-Bearing Measurement of the Hind Limb

The data of OA-induced right hind limb were recorded using an incapacitance meter (IITC Life Science Inc., Burbank, CA, USA) at 0–24 d after OA induction in the SD rats. The average weight balance of each limb was analyzed as follows:

Weight-bearing ratio (%) = (weight on right hind limb/weight on left and right hind limbs) × 100.

### 4.7. Assessment of Chondral Degradation

After sacrifice, the cartilage was removed from the right knee and evaluated by macroscopic scoring for articular cartilage erosion (Table 4) [54,55]. The right knee was photographed using a camera (Sony Corp., Tokio, Japan).

### 4.8. Serum Analysis of MIA-Induced OA Animal Model

Whole blood was extracted after sacrificing the OA rats. The whole blood was centrifuged (10 min, 4000 rpm) for isolated the serum. Multiplex analysis was performed with IL-1β and IL-6 using a MultiAnalyte Kit (R&D Systems Inc., Minneapolis, NE, USA) to obtain the cytokine measurements in the serum, and the cytokines were investigated with Luminex analyzer (Luminex Co., Austin, TX, USA). All analyses were performed according to the manufacturer’s instructions.

### 4.9. Cell Culture

RAW264.7 cells were purchased from the Korean Cell Line Bank (Seoul, Republic of Korea). The cells were cultured in DMEM containing 10% FBS and 5% penicillin–streptomycin (Gibco BRL, Billings, MT, USA) in a 5% CO_2_ incubator (Thermo Fisher Inc., Seoul, Republic of Korea).

### 4.10. Analysis of Cell Viability and Nitric Oxidate (NO) Production

The RAW264.7 cells were grown for 24 h. After one day, the cells were treated with GP (10–300 µg/mL) and LPS (500 ng/mL) for 24 h. Dexamethasone (DEX 1; Sigma, St. Louis, MO, USA) was used as a positive control. Cell viability was measured using EzCytox (DoGenBio, Seoul, Republic of Korea) following the manufacturer’s protocol. The NO production assay was performed using a NO assay kit (Sigma). The NO concentration assay was performed according to manufacturer’s protocol. This experiment was performed in triplicate.

### 4.11. Measurement of mRNA Expression Levels Using qRT-PCR

RNAs was extracted from the LPS-treated RAW264.7 cells and right knee joint cartilage of the OA rats. An RNA Extraction Kit (Bioneer, Daejeon, Republic of Korea) was used to extract the total RNA from the OA-induced cartilage tissue and LPS-treated RAW264.7 cells. The extracted RNA was converted to cDNA using a conversion kit (Bioneer, Daejen, Republic of Korea), and mRNA expression was determined according to the manufacturer’s protocol (Bioneer). The results were analyzed and compared with those of the CON group. The primer sequences are shown in Table 5 and Table 6.

### 4.12. Analysis of Protein Expression by Western Blotting

The protein expression level of IL-1β, IL-6, NOS2, MMP-3, MMP-8, MMP-13, and GAPDH was analyzed through the western blotting assay. Total protein was extracted from the LPS-induced RAW264.7 cells and OA rat right knee cartilage tissues using Radio-Immunoprecipitation Assay solution (Cell Signaling Technology Inc., Danvers, MA, USA) with Protease Inhibitor (Sigma, St. Louis, MO, USA) and a homogenizer (Nissei Corp., Toyama, Japan). Equal amounts of the protein samples were loaded onto SDS-PAGE gels for electrophoresis, and the isolated protein samples were transferred to membranes for 45 min at 100 V. The membranes were incubated with a blocking buffer solution (BioRad, Hercules, CA, USA) for 15 min at room temperature. And then, the membranes were washed 7 times with TBST buffer, and the 1st antibodies (IL-1β, IL-6, NOS2, MMP-3, MMP-8, MMP-13, and GAPDH) were attached for 24 h at 4 °C. All the antibodies were provided by Abcam Inc., (Cambridge, UK), Proteintech Group, Inc., (Rosemont, IL, USA), Boster Inc., (Pleasanton, CA, USA) and Cell Signaling Technology, Inc., (Danvers, MA, USA) (Table 7). The membranes were attached to the 2nd antibody for 1 h at room temperature and then reacted with ECL solution (Bio-Rad, Inc., Hercules, CA, USA). Western blotting was performed using ChemiDoc (Azure Biosystems, Dublin, CA, USA).

### 4.13. Statistical Analysis

One-way ANOVA and Dunnett’s post hoc test were used to analyze the data using GraphPad Prism (version 9.0; GraphPad Software, San Diego, CA, USA). Statistical significance was verified at *p* < 0.05, with measurements indicated as mean ± standard error.

## 5. Conclusions

This study demonstrated that GP exerted a multifaceted effect of analgesia, functional improvement, and chondroprotection in animal models of OA, supported by its potent anti-inflammatory activity against a broad spectrum of targets. In multiple in vivo and in vitro models, these effects were consistent and significant relative to the active controls, and were repeatedly observed. Furthermore, the targets for which anti-inflammatory activity was observed were highly implicated in the inflammatory pathogenesis of OA. Therefore, GP may be a promising candidate for DMOADs, a new class of drugs that inhibit the progressive pathology of OA, and is worthy of further investigation. However, to refine this hypothesis, a clearer understanding of the active ingredients and mechanisms of action of GP, as well as its effects on a wider range of OA pathologies beyond the scope of this study, need to be demonstrated through multiple experiments.

## Figures and Tables

**Table 1:** Conditions for HPLC analysis.

	Condition
Colum	Agilent Zorbax Extend C18 column (250 mm × 4.6 mm, 5 μm; Agilent, Santa Clara, CA, USA)
Mobile phase	(A) 0.1% Phosphoric acid, (B) acetonitrile
Flow rate	0–5 min, 20–20%; 5–10 min, 20–40%; 10–15 min,40–40%; 15–25 min, 40–80%; 25–35 min, 80–20%; 35–40 min, 20–20% (B)
Injection volume	1.0 mL/min
Detection wavelength	203 nm
Temperature	30 °C

**Table 2:** AAW model design.

Group	AAW Inducer (10 mL/kg, i.p.)	Sample (10 mL/kg, P.O.)	n
CON	0.7% acetic acid	DW	8
IBU 200	0.7% acetic acid	ibuprofen 200 mg/kg	8
GP 200	0.7% acetic acid	GP 200 mg/kg	8
GP 600	0.7% acetic acid	GP 600 mg/kg	8

**Table 3:** MIA-induced OA model design.

Group	OA Inducer (50 μL, Intra-Articular)	Sample (10 mL/kg, P.O.)	n
Sham	Saline	DW	9
CON	MIA 40 mg/mL	DW	9
INDO 3	MIA 40 mg/mL	indomethacin 3 mg/kg	9
GP 300	MIA 40 mg/mL	GP 300 mg/kg	9

**Table 4:** Macroscopic scoring of cartilage degradation.

Grade	Cartilage Appearance
0	Normal knee cartilage surface
1	The surface of the knee is slightly yellow discolored or exhibits some bruising
2	Erosion that reaches the superficial or middle layer of cartilage
3	Extensive erosion to subchondral bone
4	Large-scale erosion with massive exposure of subchondral bone

**Table 5:** Primer sequence of OA rat model.

Gene Name	Amplicon Size	Accession No.	Direction	Sequence
*IL-1β*	196 bp	M98820	F	AACTCAACTGTGAAATAGCAGC
			R	TCCACAGCCACAATGAGTG
*IL-6*	146 bp	M26744	F	TCCGCAAGAGACTTCCAGC
			R	CCTCCGACTTGTGAAGTGG
*NOS2*	151 bp	NM_012611	F	AGTCAACTACAAGCCCCACG
			R	GCAGCTTGTCCAGGGATTCT
*Ptger2*	120 bp	AF302686	F	TGTGTGTACTGTCCGTCTGC
			R	CAGGGATCCAGTCTCGGTGT
*MMP-1*	102 bp	NM_001134530	F	AACTTGGGTGAAGACGTCCA
			R	TCCTGTCACTTTCAGCCCAA
*MMP-3*	182 bp	NM_133523	F	GTACGGCTGTGTGCTCATCC
			R	TCAGCCCAAGGAACTTCTGC
*MMP-8*	156 bp	NM_022221	F	TCTGTTCTTCTTCCACACACAG
			R	GCAATCATAGTGGCATTCCT
*MMP-13*	198 bp	NM_133530	F	ACCTTCTTCTTGTTGAGTTGGA
			R	CTGCATTTCTCGGAGTCTA
*GAPDH*	135 bp	AF106860	F	CTTGTGACAAAGTGGACATTGTT
			R	TGACCAGCTTCCCATTCTC

**Table 6:** Primer sequence of LPS-treated RAW264.7 cells.

Gene Name	Amplicon Size	Accession No.	Direction	Sequence
*IL-1β*	132 bp	NM_008361	F	CCAGCTTCAAATCTCGCAGC
			R	GTGCTCATGTCCTCATCCTGG
*IL-6*	110 bp	NM_031168	F	CACTTCACAAGTCGGAGGCT
			R	CAAGTGCATCATCGTTGTTC
*NOS2*	160 bp	NM_001313922	F	ACCAAGATGGCCTGGAGGAA
			R	CCGACCTGATGTTGCCATTG
*Ptger2*	160 bp	NM_008964	F	CTGGTAACGGAATTGGTGC
			R	TGGCCAGACTAAAGAAGGTC
*COX-2*	144 bp	NM_011198	F	ATCCATGTCAAAACCGTGGG
			R	TTGGGGTGGGCTTCAGCAG
*TNF-α*	140 bp	NM_013693	F	GAGAAGTTCCCAAATGGCCT
			R	AGCCACTCCAGCTGCTCCT
*MMP-3*	91 bp	NM_010809	F	AAGTTCCTCGGGTTGGAGAT
			R	ACCAACATCAGGAACACCAC
*MMP-13*	161 bp	NM_008607	F	AACCAAGATGTGGAGTGCCT
			R	GACCAGACCTTGAAGGCTTT
*GAPDH*	208 bp	NM_001411840	F	ATGGTGAAGGTCGGTGTG
			R	GCCGTGAGTGGAGTCATAC

IL: interleukin, NOS: nitric oxide synthase, Ptger2: prostaglandin E receptor 2, MMP: matrix metalloproteinase, GAPDH: Glyceraldehyde 3-phosphate dehydrogenase.

**Table 7:** Antibodies.

Antibody	Company	Cat No.
IL-1β	Abcam	Ab283818
IL-6	Abcam	Ab259341
NOS2	Boster	A00368-1
Ptger2	Boster	M04963
MMP-3	Abcam	Ab52915
MMP-8	Abcam	Ab81286
MMP-13	Abcam	Ab39012
GAPDH	Cell Signaling Tech	#2118

## Data Availability

All data from this study are included in the main body of the article.

## Supplementary Materials

The following supporting information can be downloaded at: https://www.mdpi.com/article/10.3390/ijms25179594/s1.
