# Supplementary material for: Investigating the Anti-Inflammatory, Analgesic, and Chondroprotective Effects of Gynostemma pentaphyllum (Thunb.) Makino in Osteoarthritis: An In Vitro and In Vivo Study
Source: Int J Mol Sci. 2024 Sep 4;25(17):9594. doi: 10.3390/ijms25179594 (PMC11395165; doi:10.3390/ijms25179594)

<GAPDH: cartilage & cell>

1: sham
2: CON
3: INDO 3
4: GP 300

5: NT
6: CON
7: DEX 1
8: GP 30
9: GP 100
10: GP 300

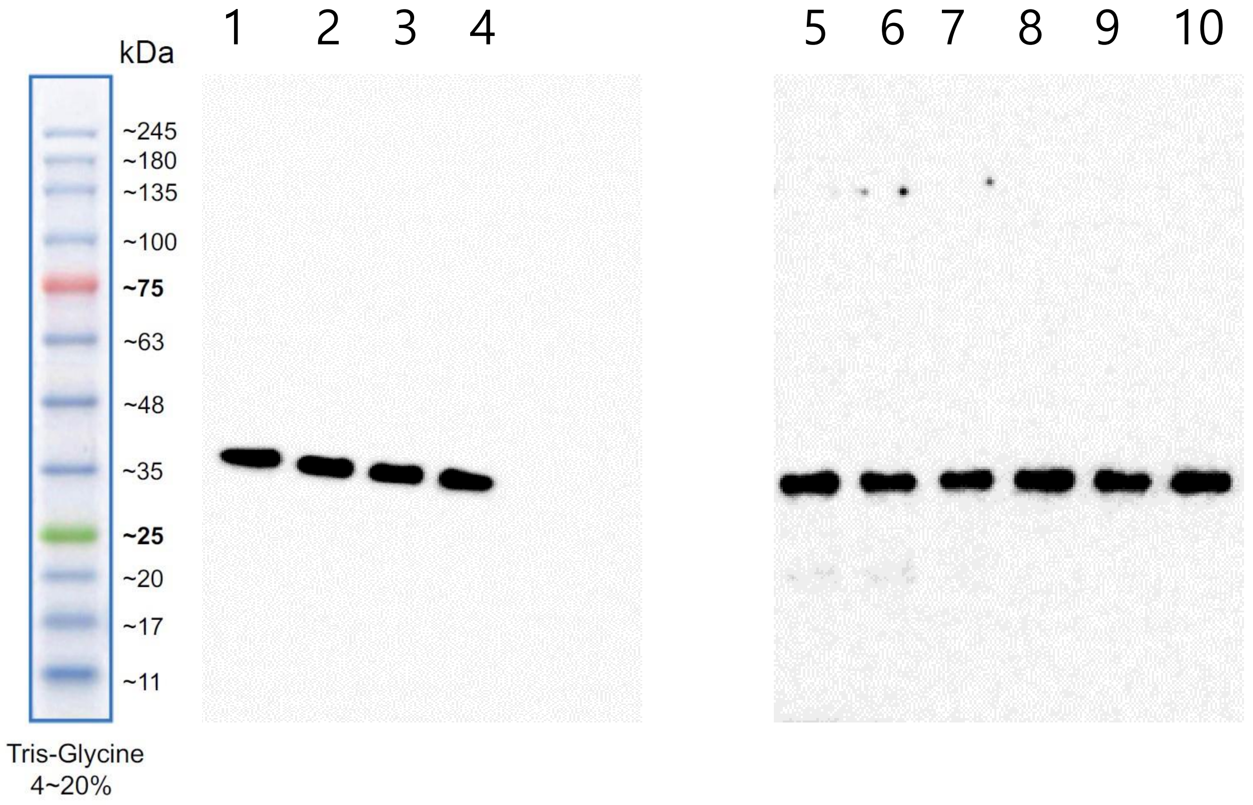

<NOS2: cartilage & cell >

1: sham
2: CON
3: INDO 3
4: GP 300

5: NT
6: CON
7: DEX 1
8: GP 30
9: GP 100
10: GP 300

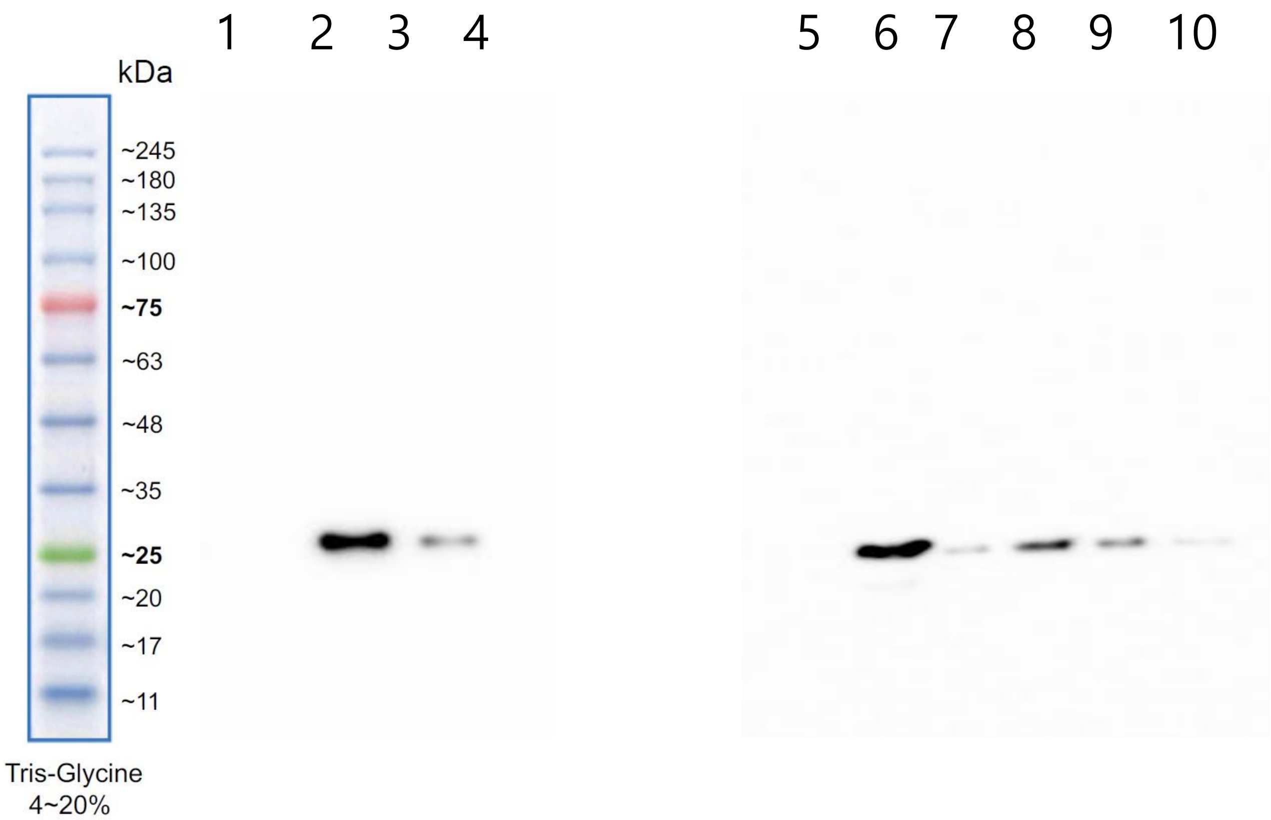

<MMP3 : cartilage & cell >

1: sham
2: CON
3: INDO 3
4: GP 300

5: NT
6: CON
7: DEX 1
8: GP 30
9: GP 100
10: GP 300

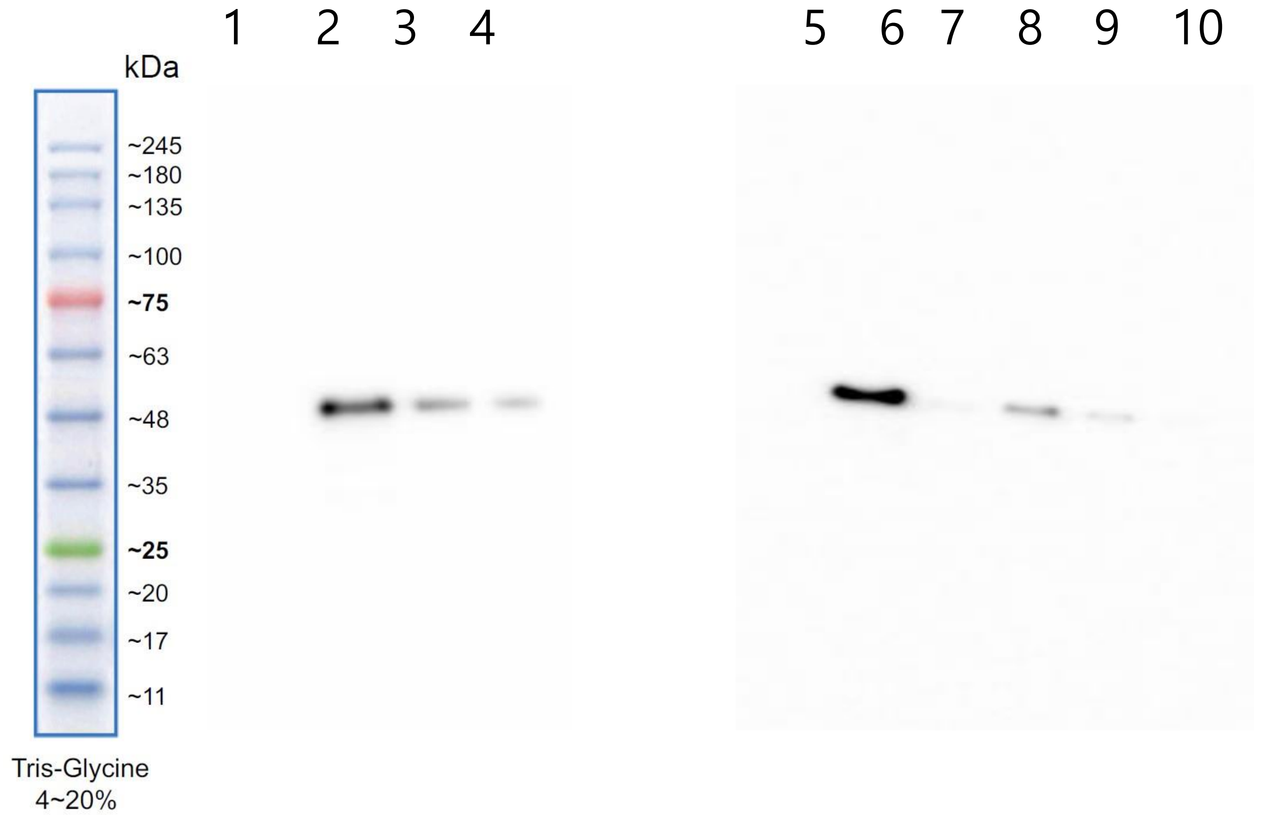

<MMP8 : cartilage & cell >

1: sham
2: CON
3: INDO 3
4: GP 300

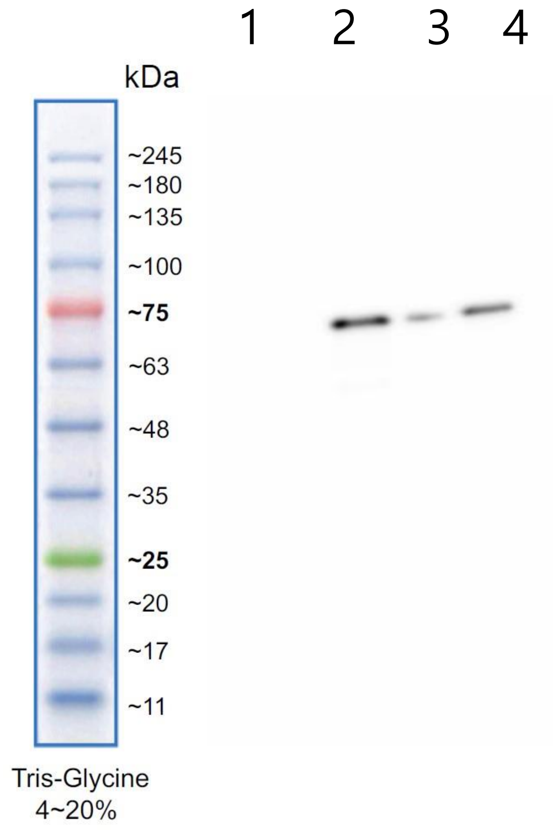

<IL-1 beta : cartilage & cell >

1: sham
2: CON
3: INDO 3
4: GP 300

5: NT
6: CON
7: DEX 1
8: GP 30
9: GP 100
10: GP 300

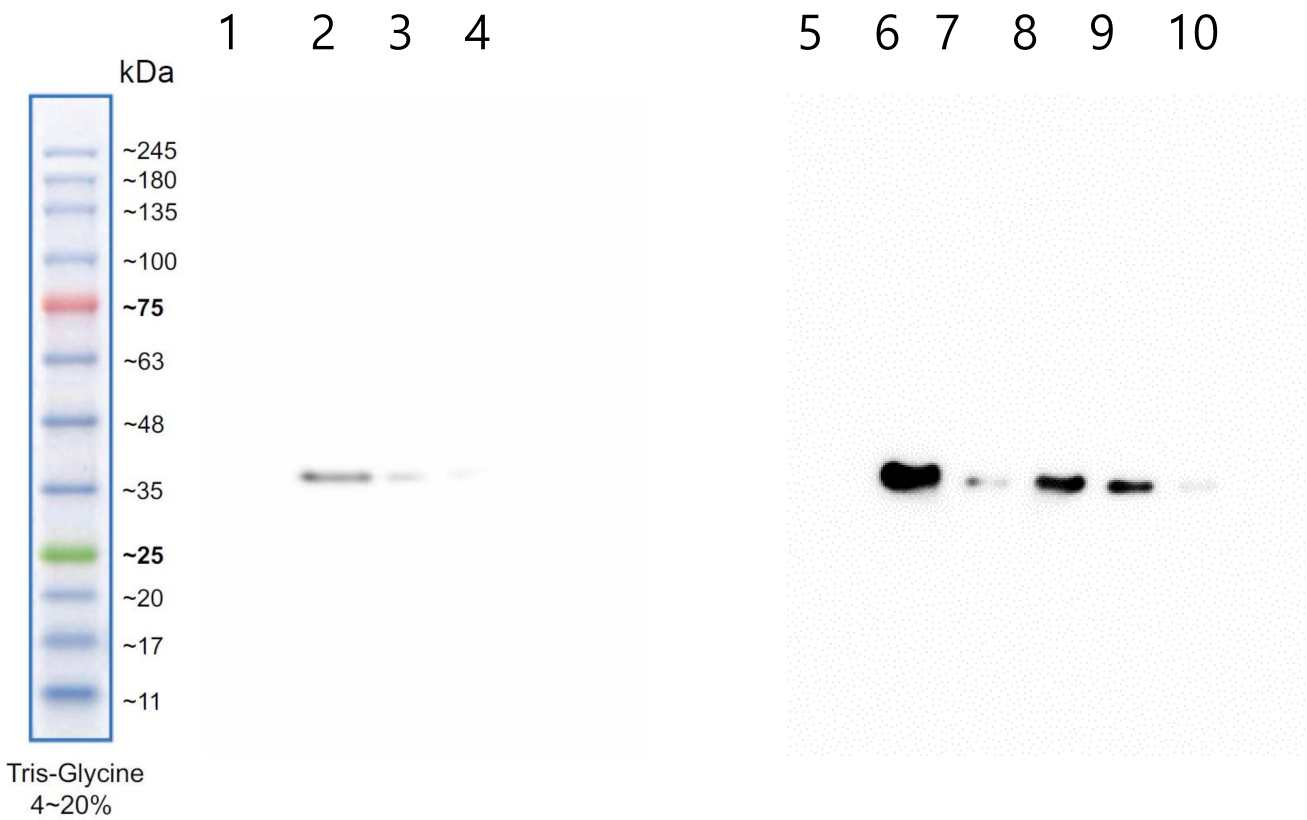

<IL-6 : cartilage & cell >

1: sham
2: CON
3: INDO 3
4: GP 300

5: NT
6: CON
7: DEX 1
8: GP 30
9: GP 100
10: GP 300

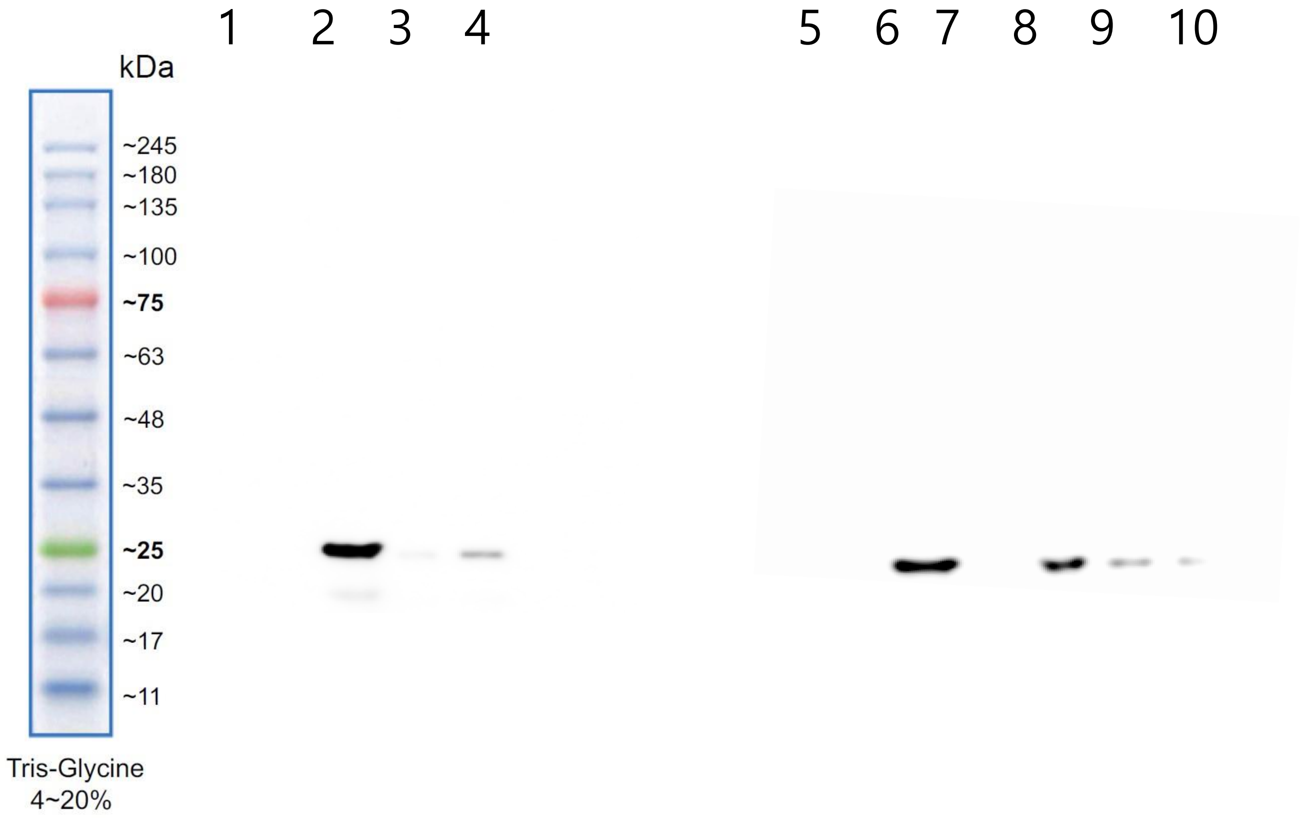

<ptger2 : cartilage & cell >

1: sham
2: CON
3: INDO 3
4: GP 300

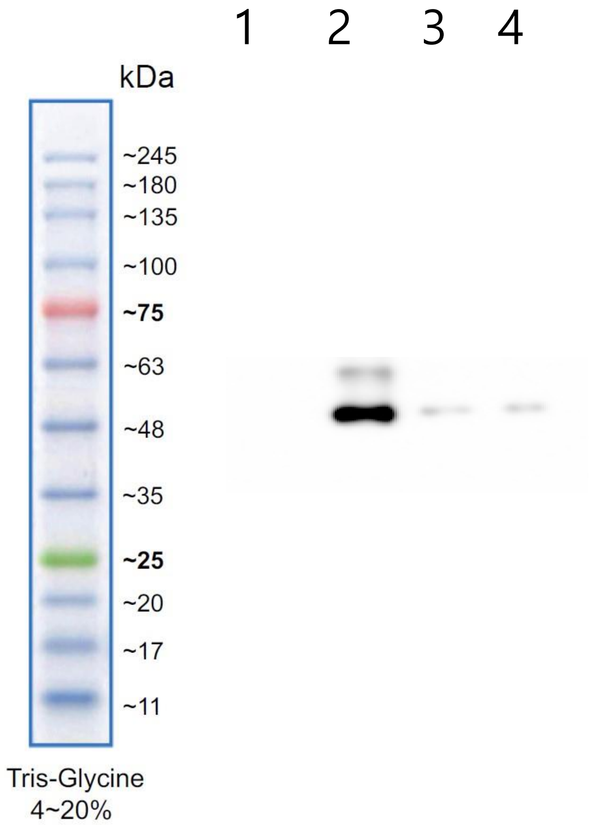

<MMP13 : cartilage & cell >

1: sham
2: CON
3: INDO 3
4: GP 300

5: NT
6: CON
7: DEX 1
8: GP 30
9: GP 100
10: GP 300

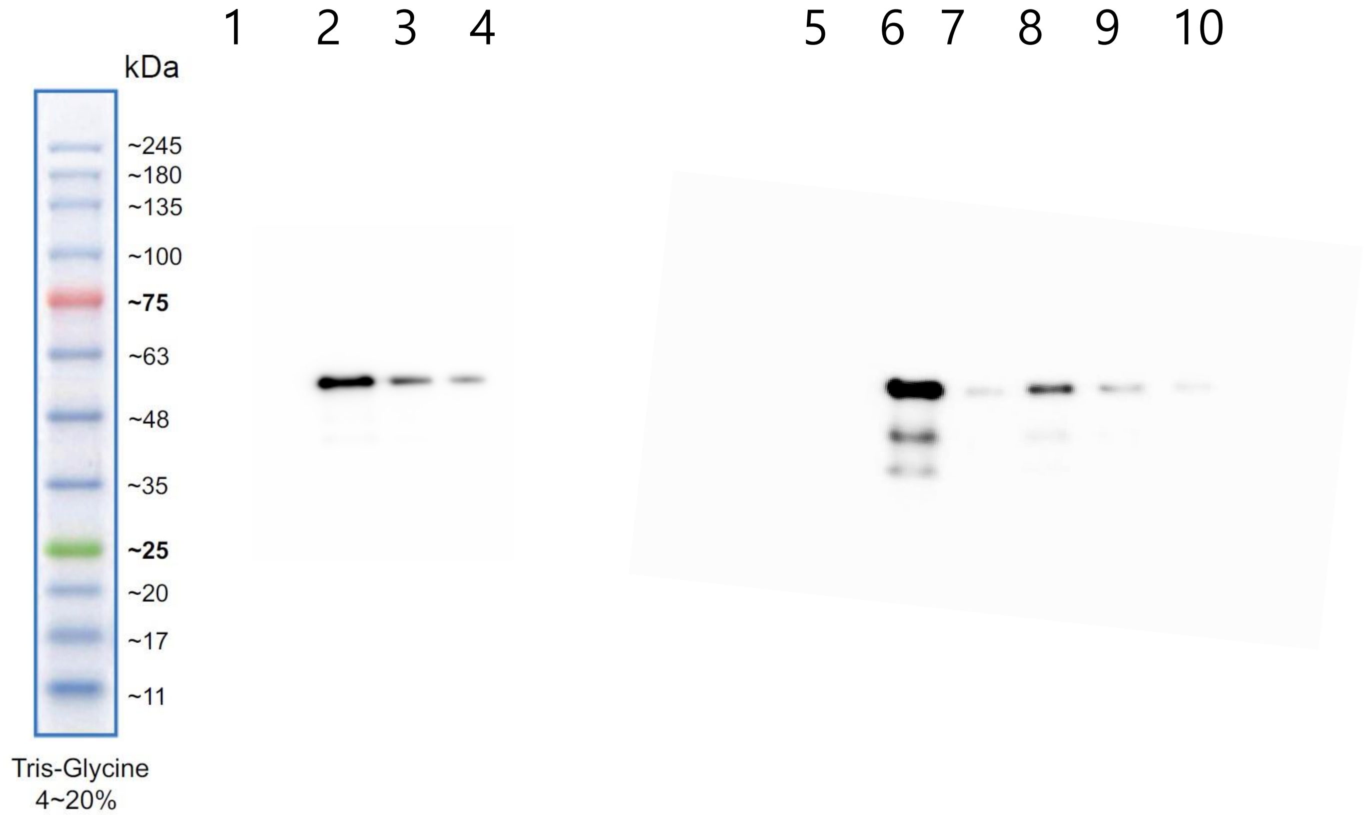

Supplement: Supplementary file 1 [file ijms-25-09594-s001.zip › ijms-3138978-supplementary.pdf]
